# Alcohol septal ablation in hypertrophic cardiomyopathy

**DOI:** 10.21542/gcsp.2018.30

**Published:** 2018-08-12

**Authors:** Juan José Santos Mateo, Juan R. Gimeno

**Affiliations:** 1 Hospital Virgen del Castillo, Yecla, Murcia, Spain; 2 Hospital Universitario Virgen de La Arrixaca, El Palmar, Murcia, Spain

## Abstract

Alcohol septal ablation (ASA) has become an alternative to surgical myectomy in obstructive hypertrophic cardiomyopathy since it was first introduced in 1994 by Sigwart. The procedure alleviates symptoms by producing a limited infarction of the upper interventricular septum, resulting in a decrease in left ventricular outflow tract (LVOT) gradient. The technique has been improved over time and the results are comparable with those of myectomy. Initial concerns about long-term outcomes have been largely resolved. In this review, we discuss indications, technical aspects, clinical results and patient selection to ASA.

## Introduction

Hypertrophic cardiomyopathy (HCM) is the most common inheritable cardiac disease, with a prevalence of 1 in 500 persons. It is characterized by marked hypertrophy of the myocardium that provokes diastolic dysfunction, left ventricular outflow tract obstruction and an increased risk of arrhythmias. In addition, it is highly heterogeneous. Most individuals with HCM have near-normal life expectancy, and they remain asymptomatic throughout life. On the other hand, some patients develop symptoms of heart failure, angina, syncope or even sudden cardiac death caused by different mechanisms.^1,2^

**Figure 1.**
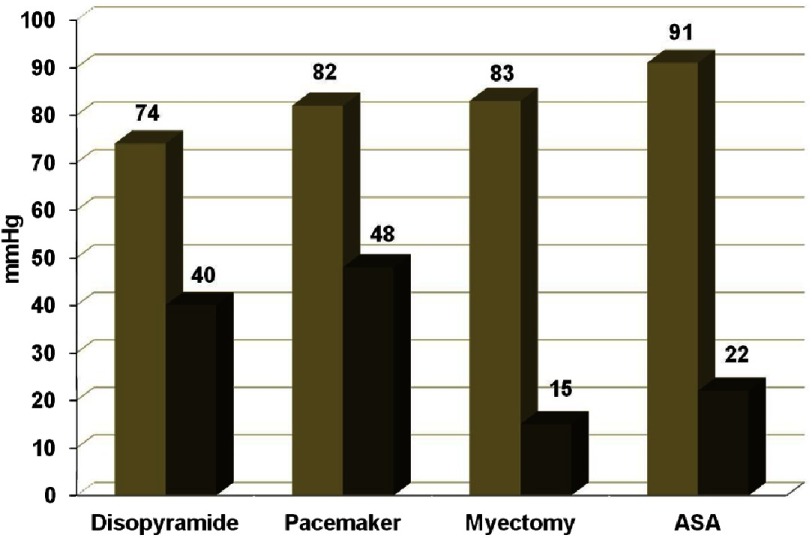
Reductions in the mean resting LVOT gradient in different treatments. Light grey bars represent pre-treatment gradients. Black bars represent post-treatment gradients.

Nearly two-thirds of patients with HCM have a significant gradient across the left ventricular outflow tract (LVOT) at rest, during provocation manoeuvres or exercise, and are classified as obstructive HCM. The main treatment in these patients is negative inotropic drugs, such as beta-blockers, calcium channel antagonist and disopyramide. Between 5–10% remain symptomatic and need septal reduction therapy, either surgical septal myectomy or alcohol septal ablation^3–6^ (Figure 1).

Brugada et al. were the first to use alcohol into the septal branch of left anterior coronary artery to treat refractory ventricular tachycardia^7^. Sigwart et al.^8^ and Kuhn et al.^9^ subsequently reported on reduction of systolic wall motion in HCM by temporary balloon occlusion of the coronary artery. Later, Sigwart introduced catheter-based delivery of absolute alcohol to provoke a small septal infarction as an alternative to surgical myectomy^10,11^.

### Pathophysiology of obstruction

Obstruction to left ventricular outflow results from the combined effect of severe septal hypertrophy and abnormalities of the mitral valve apparatus. Ejection flow drags elongated and abnormally positioned anterior mitral leaflet into LVOT. Following this, the LVOT orifice is narrowed further and greater obstruction to flow develops. Also, co-aptation of mitral leaflet is distorted and appearing dynamic mitral regurgitation, which plays an important role in symptoms.^3–5^ LVOT obstruction has several physiopathological consequences, including reduction of cardiac output, diastolic dysfunction, secondary mitral regurgitation, and myocardial ischemia. These factors are related with symptoms of dyspnoea, chest pain, presyncope and syncope, and are associated with a worse prognosis^3,5^.

### Indications for septal reduction therapy

Septal reduction therapy should be considered in patients with an LVOTO gradient ≥50 mmHg, moderate to severe symptoms (New York Heart Association (NYHA) functional Class III–IV) (Recommendation I C) and/or recurrent exertional syncope (Recommendation IIa C) in spite of maximally tolerated drug therapy^1,2,5^.

This intervention should be performed by experienced operators defined as an individual operator with a cumulative case volume of at least 20 procedures (AHA/ACC guidelines) or a minimal caseload of 10 ASA or myectomies (ESC guidelines). Some studies have shown a relationship between results and hospital volumes^1,2^.

Kim et at^12^ demonstrated that 60% of centres in the U.S. had performed <10 myectomies during the 9-year study period. This is important as the low-volume centres were found to have higher in-hospital mortality rates (15.6% vs. 3.8% *p* < 0.001), need for permanent pacemaker (10.0% vs. 8.9%; *P* < 0.001), and bleeding complications (3.3% vs. 1.7%; *P* < 0.001) after septal myectomy compared with high-volume centres. For ASA, 67% of centres performed <10 procedures, but ASA procedures in low-volume centres were not associated with worse outcome. In contrast, a recent study^13^ based on Euro-ASA registry cohort showed significant association between institutional experience and an almost two-fold lower incidence of periprocedural major adverse events, and significantly better efficacy and safety in long-term follow up after an institutional experience was achieved.

As there are no randomized trials comparing surgery and ASA, guidelines are based on observational studies. The 2011 AHA/ACC guidelines consider septal myectomy as the gold standard technique for septal reduction therapy, and advise against performing ASA in younger patients, severe septal thickness (>25–30 mm), mid-ventricular obstruction and in the presence of concomitant cardiac disease. They specifically recommend ASA in the elderly and in patients with significant comorbidity that increases surgical risk or when patients refuse open-heart surgery.^2,14^ ESC guidelines^1^ do not give priority to one technique over another, and suggest an individual assessment with an experienced multidisciplinary team.

Recently, a small study^15^ has evaluated long-term outcomes of mildly symptomatic patients (NYHA II) treated with ASA. The 30-day mortality after ASA was lower than previously reported (0.6%) and annual all-cause mortality rate was similar to the general population. After almost 5 years of follow-up, 69% remained in NYHA I class and haemodynamic improvement remained similar as at the beginning. In addition, some studies^16,17^ have shown good results in periprocedural mortality rate (0.3% vs. 2%, *p* = 0.03), pacemaker implantation (8% vs 16%, *p* < 0.001), NYHA status (95% NYHA I-II), lower annual mortality rates (1% vs 5%, *p* < 0.01) with similar arrhythmic event rates (1%) in younger ages (<50 years). These studies could be the initial evidence to broaden the indication for ASA to younger patients, but these data should be confirmed before in larger studies.

ASA is controversial in children, adolescents and young adults as there are no long-term data on the late effects of a myocardial scar in these groups, and because the technical difficulties and potential hazards of the procedure in smaller children and infants are greater. Anecdotal cases have been reported where children were treated with ASA after unsuccessful surgical myectomy and were not candidates for heart transplantation^1,2,18^.

**Figure 2.**
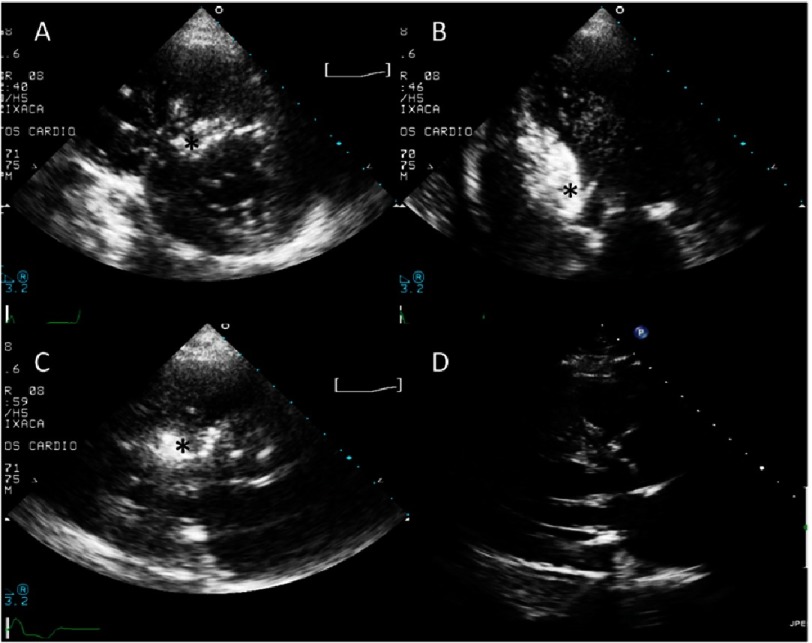
Contrast echocardiography. Multiple projections: A. Paraesternal short axis. B. Apical four-chamber. C. Paraesternal long axis. * Opacification of the targeted area of the septum. D. Paraesternal long axis after septal ablation.

### Procedure

At most centres, the technique performed is that proposed by Faber et al.,^19^ which uses myocardial contrast echocardiography^11^. First, it is necessary to have two arterial access sites for coronary guide-catheter and pigtail catheter, and one venous access site (usually femoral or jugular) for pacing electrode. Before the ablation, a diagnostic catheterization is performed to measure the left ventricular outflow tract and to exclude coronary artery disease and select a potential target septal artery. The outflow pressure gradient is usually measured by catheterization and Doppler echocardiography at rest and after induction of extrasystoles with a pigtail catheter or programmed stimulation utilizing the temporary pacemaker^3,4,6^.

Since almost 50% of patients develop a transient complete heart block, implantation of a temporary pacemaker lead is mandatory in all patients without a previous permanent pacemaker or ICD^11^. Using internal jugular vein access site and conventional permanent active fixation pacing electrode connected to a permanent sterilized pacemaker generator allows improving electrode stability, patient mobility and minimizing the risk of cardiac perforation^20,21^.

A guide-wire is advanced into the target septal artery; afterwards an over the wire balloon is advanced into the target septal artery. This is inflated and isolates the septal artery from the other coronary territories. Selective angiography of the target septal branch through the inflated balloon catheter should document the adequate sealing of the septal branch. Consequently, echocardiographic contrast agent is injected through the balloon catheter with continuous echocardiographic screening. An obvious opacification of the area of the septum involved in the contact point for SAM will be seen if the artery is the correct choice. Multiple projections are required to ensure the correct distribution. Myocardial contrast echocardiography allows higher success rates despite lower infarct sizes, in turn reducing complication rates^22,23^ (Figure 2).

A small volume (1–3 mL) of absolute alcohol is injected slowly in small increments through the central lumen of the balloon catheter under continuous fluoroscopic, haemodynamic, echo and electrocardiographic control. The quantity of injected alcohol should be determined by the septal thickness (1 ml/10 mm). Analgesia should be given for control of chest pain. Balloon occlusion should be maintained for at least 10 mins. After deflating the balloon catheter, an angiogram is performed to confirm complete occlusion of the septal branch and normal flow in the left anterior descending artery. In different registries <5% ASA procedures have been aborted for lack of an appropriate septal branch^24^.

The gradient commonly decreases during the procedure, though the beneficial effect is the consequence of a slow process of fibrosis and ventricular remodelling that is not achieved until some months later. The relationship between the acute results and the long-term benefits is poor.

Patients are observed in the cardiac intensive care unit for 24–72 hours. Cardiac enzyme measurements every 6 to 8 hours allow documentation of peak creatine kinase or troponin value. If no complete heart block is present at that time, temporary pacing wires can be removed. Hospital stay is usually 5 days if no complication is observed (although some centres advocate up to 1 week), and this is predominantly to observe for late complete heart block.

**Table 1:** Outcomes of main studies of ASA.

Study	Period	*n*	Mean follow up	Mean age (years)	30-days mortality (%)	Periprocedure VA (%)	PM (%)	Redo-ASA (%)	Long-term mortality (annual/ %)	SCD (annual/%)	Mean LVOT gradient (pre/post ASA)	NYHA >III (%)
Steggerda, 2014^30^	1981–2010	161	5.1	59	1.2	2.5	7	6	1.5	–	101/19^*^	16
Vriesendorp, 2014^31^	1990–2012	321	7.5	58	1.6	3.1	–	9.7	1.9	0.96	97/10	–
Veselka, 2014^32^	1998–2013	178	4.8	58	0.6	3	8	3	2.1	0.3	68/20	13
Veselka, 2016^24^	1996–2015	1275	5.7	58	1	1.6	12	7	2.4	1.16	67/16	11
Liebregts, 2015^25^	1963–2013	2013	6.2	56	1	2.2	10	8	1.5	0.4	–	–
Singh, 2016^26^	1972–2015	805	2.9	49	0.9	–	17	9	1.8	–	78/19	<10

**Notes.**

SCD Sudden cardiac death PM Pacemaker ASA Alcohol septal ablation LVOT Left ventricle outflow tract NYHA New York Heart Association VA Periprocedure ventricular arrhythmia, including sustained ventricular tachycardia, ventricular fibrillation and ICD discharges

* Provoked gradient. Baseline gradient 32/10mmHg.

## Results

### Periprocedural complications and long-term outcomes

Complications are rare with experienced operators. The 30-day mortality for septal ablation is now <1%, with severe cardiac events occurring in <2% of patients.

The most common complication is the need for permanent pacemaker. The risk in large multicentre observations remains around 10-12%, more than twice the risk of permanent pacemaker implantation compared with those who undergo myectomy.^24–27^ Patients with first-degree AV block and those with LBBB are at high risk of persistent advanced block during ASA; thus, the implantation of a permanent pacemaker prior to procedure is highly recommended^11^. Higher doses of alcohol are associated with a higher risk of heart block and subsequent pacemaker requirement; doses of alcohol ranging between 1.5 and 2.5 mL probably represent the optimum balance between efficacy and safety for most patients^24^. In addition, there is a reduction in pacemaker implantation with increased operator volume^3,12,13^. Other rare complications include coronary artery dissection, ventricular fibrillation, cardiac tamponade, cardiogenic shock, pulmonary embolism and bradyarrhythmias.

Due to iatrogenic myocardial infarction, the potential for proarrhythmia has been a concern for ASA. The work of ten Cate et al.^28^ reported an annual rate of cardiovascular death or ICD discharge to be 5.2-fold higher in the ASA group than in the myectomy group. However, no study has reproduced these results. The likely reason for these outcomes is that patients included received large volumes of alcohol (4.5 ml) and the goal was to achieve resolution of the obstruction in the laboratory^11,29^. Later published studies do not indicate an increase in incidence of ventricular arrhythmias during follow–up^24–26,30^. In the Euro-ASA registry^24^ only a few patients experience early post-procedural ventricular arrhythmias (1.6%), and the rate of sudden mortality events was 1% per year. Annual sudden cardiac death rates following ASA were also found to be similar to those in post-myectomy patients, ranging from 0.4% to 1.3%.^5^ In addition, the survival of ASA-treated patients was found to be comparable to those myectomy-treated patients and patients with non-obstructive HCM^31^, and it was comparable to the expected survival for age and sex general population (Survival free of all-cause mortality at 1, 5, and 10 years was 97%, 92% and 82% respectively) (Table 1).

**Table 2:** Considerations for selection of septal reduction therapies.

Septal myectomy	Septal ablation
Patient choice (immediate results)	Patient choice (less invasive and shorter recovery)
Concomitant cardiac disease	High surgical risk (comorbilities)
Longest follow-up data	Relatively shorter follow-up evidence
Expertise limited to few HCM centres	More reproductive results between centres
Massive hypertrophy	Mild-moderate hypertrophy (16–25 mm)
Mid-ventricular obstruction	
Younger patients	Elderly patients
	Cost of double risk of pacemaker and reinterventions.

### Treatment efficacy

The reduction of the gradient is observed immediately after surgical myectomy, whereas the benefits of ASA are delayed, often for more than 6 months after alcohol injection. Both myectomy and ASA are followed by a process of cardiac remodeling that involves the reduction of the thickness in other segments and in the size of the left atrium, a consequence of the haemodynamic improvement achieved.

Two meta-analyses showed a slightly higher LVOT gradient after ASA compared with myectomy.^25,26^ However, no significant differences were found in NYHA functional class, peak oxygen consumption and exercise capacity at late follow-up between the 2 procedures. The median percentage of patients remaining in NYHA functional class III/IV was 8% after ASA and 5% after myectomy ( *p* = 0.43), and the reduction in LVOT gradient was 71% after ASA and 77% after myectomy ( *p* = 0.63).^25,26^ The benefit of ASA in older patients is similar to that in younger patients.^16,17^ On the other hand, the incidence of additional septal reduction therapy was 7.7% following ASA compared with 1.6% following myectomy.^25^

Briefly, no significant difference in symptom relief was noted between the two approaches. ASA was as safe as myectomy regarding SCD, short-term, and long-term mortality, although is associated with more than twice the risk of permanent pacemaker implantation and a 5 times higher risk of the need for additional septal reduction therapy.

### Patient selection

Multiple studies have shown a high success rate and low complication rate with both septal myectomy and ASA, leading to excellent reduction in outflow tract obstruction and sustained improvement in symptoms. The choice of procedure is dependent on many factors including the expertise and availability of the operators, the presence of concomitant cardiac problems, accompanying medical comorbidities, and patient choice (Table 2). Candidates for this treatment should be evaluated by a team with expertise in the diagnosis and management of patients with HCM, and both procedures should be performed by experienced operators.

## Conclusion

ASA has become an alternative to surgical myectomy that may be considered for many patients. Data indicates that functional and haemodynamic success of ASA is high and similar to that of surgery. Benefits of ASA in comparison to myectomy include shorter hospital stay, less pain, and avoidance of complications associated with surgery and cardiopulmonary bypass. Despite being widespread, the procedure should only be performed by experienced operators and on carefully selected patients.

## References

[1] Elliott PM, Anastasakis A, Borger MA, Borggrefe M, Cecchi F, Charron P (2014). 2014 ESC guidelines on diagnosis and management of hypertrophic cardiomyopathy: the task force for the diagnosis and management of hypertrophic cardiomyopathy of the European Society of Cardiology (ESC). Eur Heart J.

[2] Gersh BJ, Maron BJ, Bonow RO, Dearani JA, Fifer MA, Link MS (2011). 2011 ACCF/AHA guideline for the diagnosis and treatment of hypertrophic cardiomyopathy: executive summary: a report of the American College of Cardiology Foundation/American Heart Association Task Force on Practice Guidelines. Circulation.

[3] Cooper RM, Stables RH (2018). Non-surgical septal reduction therapy in hypertrophic cardiomyopathy. Heart.

[4] Fifer MA, Sigwart U (2011). Controversies in cardiovascular medicine. Hypertrophic obstructive cardiomyopathy: alcohol septal ablation. Eur Heart J.

[5] Liebregts M, Vriesendorp PA, Ten Berg JM (2017). Alcohol septal ablation for obstructive hypertrophic cardiomyopathy: A word of endorsement. J Am Coll Cardiol.

[6] Nishimura RA, Seggewiss H, Schaff HV (2017). Hypertrophic obstructive cardiomyopathy: Surgical myectomy and septal ablation. Circ Res.

[7] Brugada P, de Swart H, Smeets JL, Wellens H (1989). Transcoronary chemical ablation of ventricular tachycardia. Circulation.

[8] Sigwart U, Grbic M, Essinger A, Bischof-Delaloye A, Sadeghi H, Rivier JL (1982). Improvement of left ventricular function after percutaneous transluminal coronary angioplasty. Am J Cardiol.

[9] Kuhn H, Gietzen F, Leuner C, Gerenkamp T (1997). Induction of subaortic septal ischaemia to reduce obstruction in hypertrophic obstructive cardiomyopathy. Studies to develop a new catheter-based concept of treatment. Eur Heart J.

[10] Sigwart U (1995). Non-surgical myocardial reduction for hypertrophic obstructive cardiomyopathy. Lancet.

[11] Gimeno JR, Tome MT, McKenna WJ (2012). Alcohol septal ablation in hypertrophic cardiomyopathy: an opportunity to be taken. Rev Esp Cardiol (Engl Ed).

[12] Kim LK, Swaminathan RV, Looser P, Minutello RM, Wong SC, Bergman G (2016). Hospital volume outcomes after septal myectomy and alcohol septal ablation for treatment of obstructive hypertrophic cardiomyopathy: US nationwide inpatient database, 2003–2011. JAMA Cardiol.

[13] Veselka J, Faber L, Jensen MK, Cooper R, Januska J, Krejci J (2018). Effect of institutional experience on outcomes of alcohol septal ablation for hypertrophic obstructive cardiomyopathy. Can J Cardiol.

[14] Maron BJ, Nishimura RA (2014). Surgical septal myectomy versus alcohol septal ablation: assessing the status of the controversy in 2014. Circulation.

[15] Veselka J, Faber L, Liebregts M, Cooper R, Januska J, Krejci J (2017). Outcome of alcohol septal ablation in mildly symptomatic patients with hypertrophic obstructive cardiomyopathy: A long-term follow-up study based on the euro-alcohol septal ablation registry. J Am Heart Assoc.

[16] Liebregts M, Faber L, Jensen MK, Vriesendorp PA, Januska J, Krejci J (2017). Outcomes of alcohol septal ablation in younger patients with obstructive hypertrophic cardiomyopathy. JACC Cardiovasc Interv.

[17] Liebregts M, Steggerda RC, Vriesendorp PA, van Velzen H, Schinkel AFL, Willems R (2016). Long-term outcome of alcohol septal ablation for obstructive hypertrophic cardiomyopathy in the young and the elderly. JACC Cardiovasc Interv.

[18] Cano MN, Fortunato de Cano SJ, Sousa JEMR (2010). Alcohol septal ablation after myomectomy failure solutions for unusual cases. Catheter Cardiovasc Interv.

[19] Faber L, Seggewiss H, Gleichmann U (1998). Percutaneous transluminal septal myocardial ablation in hypertrophic obstructive cardiomyopathy: results with respect to intraprocedural myocardial contrast echocardiography. Circulation.

[20] Arias MA, Puchol A, Pachon M, Jimenez-Lopez J, Rodriguez-Padial L (2012). Prolonged temporary cardiac pacing using an external permanent pacing system. Rev Esp Cardiol (Engl Ed).

[21] Kawata H, Pretorius V, Phan H, Mulpuru S, Gadiyaram V, Patel J (2013). Utility and safety of temporary pacing using active fixation leads and externalized re-usable permanent pacemakers after lead extraction. Europace.

[22] Cardim N, Galderisi M, Edvardsen T, Plein S, Popescu BA, D’Andrea A (2015). Role of multimodality cardiac imaging in the management of patients with hypertrophic cardiomyopathy: an expert consensus of the European Association of Cardiovascular Imaging Endorsed by the Saudi Heart Association. Eur Heart J Cardiovasc Imaging.

[23] Nagueh SF, Bierig SM, Budoff MJ, Desai M, Dilsizian V, Eidem B (2011). American Society of Echocardiography clinical recommendations for multimodality cardiovascular imaging of patients with hypertrophic cardiomyopathy: Endorsed by the American Society of Nuclear Cardiology, Society for Cardiovascular Magnetic Resonance, and Society of Cardiovascular Computed Tomography. J Am Soc Echocardiogr.

[24] Veselka J, Jensen MK, Liebregts M, Januska J, Krejci J, Bartel T (2016). Long-term clinical outcome after alcohol septal ablation for obstructive hypertrophic cardiomyopathy: results from the Euro-ASA registry. Eur Heart J.

[25] Liebregts M, Vriesendorp PA, Mahmoodi BK, Schinkel AFL, Michels M, ten Berg JM (2015). A systematic review and meta-analysis of long-term outcomes after septal reduction therapy in patients with hypertrophic cardiomyopathy. JACC Heart Fail.

[26] Singh K, Qutub M, Carson K, Hibbert B, Glover C (2016). A meta analysis of current status of alcohol septal ablation and surgical myectomy for obstructive hypertrophic cardiomyopathy. Catheter Cardiovasc Interv.

[27] Mestres CA, Bartel T, Sorgente A, Muller S, Gruner C, Dearani J (2018). Hypertrophic obstructive cardiomyopathy: what, when, why, for whom?. Eur J Cardiothorac Surg.

[28] ten Cate FJ, Soliman OI, Michels M (2010). Long-term outcome of alcohol septal ablation in patients with obstructive hypertrophic cardiomyopathy: a word of caution. Circ Heart Fail.

[29] Sorajja P (2017). Alcohol septal ablation for obstructive hypertrophic cardiomyopathy: A word of balance. J Am Coll Cardiol.

[30] Steggerda RC, Damman K, Balt JC, Liebregts M, ten Berg JM, van den Berg MP (2014). Periprocedural complications and long-term outcome after alcohol septal ablation versus surgical myectomy in hypertrophic obstructive cardiomyopathy: a single-center experience. JACC Cardiovasc Interv.

[31] Vriesendorp PA, Liebregts M, Steggerda RC, Schinkel AFL, Willems R, Ten Cate FJ (2014). Long-term outcomes after medical and invasive treatment in patients with hypertrophic cardiomyopathy. JACC Heart Fail.

[32] Veselka J, Krejci J, Tomasov P, Zemanek D (2014). Long-term survival after alcohol septal ablation for hypertrophic obstructive cardiomyopathy: a comparison with general population. Eur Heart J.

